# Open science challenges, benefits and tips in early career and beyond

**DOI:** 10.1371/journal.pbio.3000246

**Published:** 2019-05-01

**Authors:** Christopher Allen, David M. A. Mehler

**Affiliations:** 1 Cardiff University Brain Research Imaging Centre (CUBRIC), Wales, United Kingdom; 2 Department of Psychiatry, University of Muenster, Germany

## Abstract

The movement towards open science is a consequence of seemingly pervasive failures to replicate previous research. This transition comes with great benefits but also significant challenges that are likely to affect those who carry out the research, usually early career researchers (ECRs). Here, we describe key benefits, including reputational gains, increased chances of publication, and a broader increase in the reliability of research. The increased chances of publication are supported by exploratory analyses indicating null findings are substantially more likely to be published via open registered reports in comparison to more conventional methods. These benefits are balanced by challenges that we have encountered and that involve increased costs in terms of flexibility, time, and issues with the current incentive structure, all of which seem to affect ECRs acutely. Although there are major obstacles to the early adoption of open science, overall open science practices should benefit both the ECR and improve the quality of research. We review 3 benefits and 3 challenges and provide suggestions from the perspective of ECRs for moving towards open science practices, which we believe scientists and institutions at all levels would do well to consider.

## Abstract 2

This Perspective article offers a balanced perspective on both the benefits and the challenges involved in the adoption of open science practices, with an emphasis on the implications for Early Career Researchers.

## Introduction

Pervasive failures to replicate published work have raised major concerns in the life and social sciences, which some have gone so far as to call a ‘crisis’ ([1–6], also see [7–9]). The potential causes are numerous, well documented, and require a substantive change in how science is conducted [2,4,10–16]. A shift to open science methods (summarised in Table 1) has been suggested as a potential remedy to many of these concerns [2,4,17]. These encompass a range of practices aimed at making science more reliable, including wider sharing and reanalysis of code, data, and research materials [2,18]; valuing replications and reanalyses [2,5,6,19,20]; changes in statistical approaches with regards to power [21,22] and how evidence is assessed [23]; interactive and more transparent ways of presenting data graphically [24,25]; potentially the use of double-blind peer review [26]; and the use of formats such as preprints [27] and open access publishing. In our experience of these, the adoption of study preregistration and registered reports (RRs) is the change that most affects how science is conducted. These approaches require hypotheses and analysis pipelines to be declared publicly before data collection [2,14,28,29] (although protocols can be embargoed). This makes the crucial distinction between confirmatory hypothesis testing and post hoc exploratory analyses transparent. In the case of RRs, hypotheses and methods are peer reviewed on the basis of scientific validity, statistical power, and interest, and RRs can receive in principal acceptance for publication before data is collected [29,30]. RRs can thereby increase the chances of publishing null findings, as we demonstrate in ‘Benefit 1: Greater faith in research’. These preregistration approaches also circumvent many of the factors that have contributed to the current problems of replication [2,4,11,19]. Preregistering hypotheses and methods renders so-called hypothesizing after the results are known (HARKing) [31] impossible and prevents manipulation of researcher degrees of freedom or *p* hacking [4]. In addition, because most RR formats involve peer review prior to data collection, the process can improve experimental design and methods through recommendations made by reviewers.

**Table 1:** Open science practices. Some methods introduced or suggested by the open science community to improve scientific practices.

Resources	Sharing of code, data, research materials, and methods [2,19].
Publishing formats	Registered reports [28], preregistrations [17], exploratory reports [32], preprints [27], open access publishing [33], as well as new evaluation and peer review processes [24].
Research questions	Pursuing replications and reanalyses [2,5,6,9,19].
Methodology	Changes in statistical approaches for power [21,22], how evidence is assessed [23] and communicated [34], as well as documenting data analysis in a way that facilitates reproducing results [35].

There are promising and important reasons to implement and promote open science methods, as well as career-motivated reasons [27,36,37]. However, there are also major challenges that are underrepresented and particularly affect those who carry out the research, most commonly early career researchers (ECRs). Here, we review 3 areas of challenge posed by open science practices, which are balanced against 3 beneficial aspects, with a focus on ECRs working in quantitative life sciences. Both challenges and benefits are accompanied by suggestions, in the form of tips, which may help ECRs to surmount these challenges and reap the rewards of open science. We conclude that overall, open science methods are inevitable to address concerns around replication, are increasingly expected, and ECRs in particular can benefit from being involved early on.

## Three challenges

### Challenge 1: Restrictions on flexibility

Statistical hypothesis testing is the predominant approach for addressing research questions in quantitative research, but a point often underemphasised is that a hypothesis can only be truly held before the data are looked at, usually before the data are collected. Open science methods, in particular preregistrations and RRs, respect this distinction and require separating exploratory analyses from planned confirmatory hypothesis testing. This distinction lies at the centre of RRs and preregistrations [29,30], in which timelines are fixed, enforcing a true application of hypothesis testing but also forcing researchers to stop developing an experiment and start collecting data. Once data collection has started, new learning about analysis techniques, subsequent publications, and exploration of patterns in data cannot inform confirmatory hypotheses or the preregistered experimental design. This restriction can be exasperating because scientists do not tend to stop thinking about and thus developing their experiments. Continuous learning during the course of an investigation is difficult to reconcile with a hard distinction between confirmatory and exploratory research but may be the price of unbiased science [4,11]. Open science methods do not preclude the possibility of serendipitous discovery, but confirmation requires subsequent replication, which entails additional work. Exploratory analyses can be added after registration; however, they can and should have a lower evidential status than preregistered tests. Although this particular loss of flexibility only directly and unavoidably affects the preregistered aspects of open science, the distinction between exploratory and confirmatory enquiry is a more general principle advocated in open science [32]. Closed orthodox science simply allows for the incorporation of new ideas more flexibly, if questionably.

Informing and formulating research questions based on data exploration is recommended. Being open to and guided by the data rather than mere opinions also has many merits. However, robust statistical inference requires that the time for it is restricted to the piloting (or learning) phase. Historically, ECRs have often been provided with existing data sets and learned data analyses through data exploration. Exploratory analyses and learning are desirable but are only acceptable if explicitly separated from planned confirmatory analyses [28,29]. The common practice of maintaining ambiguity between the two can convey an advantage to the traditional researcher because failure to acknowledge the difference exploits the assumption that presented analyses are planned. Distinguishing explicitly between planned and exploratory analyses can thus only disadvantage the open researcher, because denoting a subset of analyses as exploratory reduces their evidential status. We believe, however, that this apparent disadvantage is the scientifically correct approach and is increasingly viewed as a positive and necessary distinction [2,4,28]. The restriction on flexibility imposed by explicit differentiation between exploratory and confirmatory science represents a major systemic shift in how science is understood, planned, and conducted—the impact of which is often underestimated.

The more restrictive structures of open science can result in mistakes having greater ramifications than within a more closed approach. Transparent documentation and data come with higher error visibility, and the flexibility to avoid acknowledging mistakes is lost. However, for science, unacknowledged or covered-up mistakes are certainly problematic. We therefore support the view that mistakes should be handled openly, constructively, and, perhaps most importantly, in a positive nondetrimental way [38,39]. Mistakes can and will happen, but by encouraging researchers to be open about them and not reprimanding others for them, open science can counter incentives to hide mistakes.

Besides higher visibility, mistakes can also have greater ramifications owing to the loss of flexibility in responding to them and the fixed timelines, particularly under preregistered conditions. When developing full a priori analyses, pipelines anticipating all potential outcomes and contingencies should be attempted. It is rarely possible to anticipate all contingencies, and the anticipation itself can lead to problems. For example, we have spent considerable time developing complex exhaustive analyses, which may never be used because registered preliminary assumption checks failed. Had a more flexible approach been adopted, the unnecessary time investment would likely have never been made. Amendments to preregistrations and RRs are perfectly acceptable, as are iterative studies, but such changes and additions will also take time. Beyond preregistration, the greater scrutiny that comes with open science, particularly open data and code, means that there are fewer options to exploit researcher degrees of freedom. These examples illustrate how open science researchers can pursue higher standards than closed science but can encounter difficulties and restrictions because of doing so.

#### Tips

Pilot data are essential when developing complex a priori analyses pipelines and pilot data can be explored without constraint. Make and expect a distinction between planned and exploratory analyses. Preregistered and RR experiments are likely to have a higher evidential value than closed science experiments in the future and so researchers should be encouraged to use these formats. Be open about mistakes and do not reprimand others for their mistakes, rather applaud honesty.

### Challenge 2: The time cost

There are theoretical reasons why open science methods could save time. For example, a priori analysis plans constrain the number of analyses, or reviewers may be less suspicious of demonstrably a priori hypothesis. However, in our experience, these potential benefits rarely come to fruition in the current system. The additional requirements of open and reproducible sciences often consume more time: Archiving, documenting, and quality controlling of code and data takes time. Considerably more time consuming is the adoption of preregistrations or RRs, because full analysis pipelines, piloting, manuscripts, and peer review (for RRs) must occur prior to data collection, which is only then followed by the more traditional, but still necessary, stages involved in publication such as developing (exploratory) analyses, writing the final manuscript, peer reviewing, etc. For comparison, it is usually easier and quicker (although questionable) to develop complex analyses on existing final data sets rather than on separate subsets of pilot data or simulated data, as required under preregistration. In our experience, these additional requirements can easily double the duration of a project. Data collection also takes longer in open experiments, which often have higher power requirements, particularly when conducted as RRs [2,21]. The ECR who adopts open science methods will likely complete fewer projects within a fixed period in comparison to peers who work with traditional methods. Therefore, very careful consideration needs to be given to the overall research strategy as early as possible in projects, because resources are limited for ECRs within graduate programs and post-doctoral positions. Although there are discussions around reducing training periods for ECRs [40,41], the additional time requirements of open science, and in particular of preregistration and RRs, might be seen as countermanding factors that require longer periods of continuous employment to allow ECRs to adopt open science practices. Less emphasis on moving between institutions than is currently the norm may also help alleviate these concerns by allowing for longer projects. The increased time cost, in our experience, presents the greatest challenge in conducting open science and acutely afflicts ECRs and thus may require rethinking how ECR training and research is organised by senior colleagues.

#### Tips

Preregistered, well powered experiments are preferential to those that are not. However, it should be expected and planned for that these will take substantially longer than would otherwise be the case. Where possible, researchers at all levels should take this time cost into account, whether in planning research or questions of employment and reward.

### Challenge 3: Incentive structure isn’t in place yet

Open science is changing how science is conducted, but it is still developing and will take time to consolidate in the mainstream [42,43]. Systems that reward open science practices are currently rare, and researchers are primarily assessed according to traditional standards. For instance, assessment structures such as the national Research Excellence Framework (REF) in the United Kingdom or the Universities Excellence Initiative in Germany, as well as research evaluations within universities, are yet to fully endorse and reward the full range of open science practices. Some reviewers and editors at journals and funders remain to be convinced of the necessity or suitability of open methods. Although many may view open science efforts neutrally or positively, they rarely weigh proportionally the sacrifices made in terms of flexibility and productivity. For example, reviewers tend to apply the same critical lens irrespective of when tested hypotheses were declared.

High-profile journals tend to reward a good story with positive results, but loss of flexibility limits the extent to which articles can be finessed, and it reduces the likelihood of positive results (see ‘Benefit 2’). The requirement for novelty can also countermand the motivation to perform replications, which, as recent findings indicate, are necessary [4,5,11,44]. Some journals are taking a lead in combatting questionable research practices and have signed guidelines promoting open methods [45,46]. However, levels of adoption are highly variable. Although many prestigious journals, institutions, and senior researchers declare their support for open methods, as yet, few have published using them.

Within open science, standards are still developing. At present, there is a lack of concessions over single-blind, double-blind, and open peer review [26]. Levels of preregistration vary dramatically [47], with some registrations only outlining hypotheses without analysis plans. Although this approach may guard against HARKing and be tactically advantageous for individuals, it does little to prevent *p* hacking and may eventually diminish the perceived value of preregistrations. There is also a practical concern around statistical power. High standards are admirable (e.g., *Nature Human Behaviour* requires all frequentist hypothesis tests in RRs be powered to at least 95%), but within limited ECR research contracts, they run up against feasibility constraints, partially for resource-intensive (e.g., neuroimaging, clinical studies) or complex multilevel experiments that are likely to contain low to medium effect sizes. Such constraints might skew areas of investigation and raise new barriers specifically for ECRs trying to work openly. However, developments in the assessment of evidence might alleviate some of these concerns in the future [22,23,48].

The challenges described above mean that ECRs practicing open science are likely to have fewer published papers by the time they are applying for their next career stage. Compounding this issue is the dilution of authorship caused by the move towards more collaborative work practices (although, see [49]). ECR career progression critically depends on the number of first and last author publications in high-profile journals [1,4,18]. These factors make it more difficult for ECRs to compete for jobs or funding with colleagues taking a more orthodox approach [27]. Furthermore, although senior colleagues may find their previous work devalued by failed replications, they are likely to have already secured the benefits from quicker and less robust research practices [43]. They may then expect and teach comparable levels of productivity, which has the potential to be a source of tension [50].

The trade-off between quality and quantity appears to be tipped in favour of quantity in the current incentive structure. As long as open science efforts are not formally recognised, it seems ECRs who pursue open science are put at a disadvantage compared with ECRs who have not invested in open science [42,51]. However, reproducible science is increasingly recognized and supported, as we will discuss in the next section. Overall, ECRs are likely to be the ones who put in the effort to implement open science practices and may thus be most affected by the described obstacles. We believe academics at all levels and institutions should take into account these difficulties because the move towards open and reproducible science may be unavoidable and can ultimately benefit the whole community and beyond.

To summarise, ECRs currently face a situation in which demands on them are increasing. However, the structures that might aid a move towards more open and robust practices are not widely implemented or valued yet. We hope that one consequence of the well-publicised failures to replicate previous work and the consequent open science movement will be a shift in emphasis from an expectation of quantity to one of quality. This would include greater recognition, understanding, and reward for open science efforts, including replication attempts, broader adoption of preregistration and RRs, expectation of explicit distinctions between confirmatory and exploratory analyses, and longer, continuous ECR positions from which lower numbers of completed studies are expected.

#### Tips

Early adoption of open methods and high standards requires careful planning at an early stage of investigations but doing so should place ECRs ahead of the curve as practices evolve. Be strategic with which open science practices suit your research. Persevere, focus on quality rather than quantity, and, when evaluating others’ work, give credit for efforts made towards the common good.

## Three benefits

### Benefit 1: Greater faith in research

“Science is an ongoing race between our inventing ways to fool ourselves, and our inventing ways to avoid fooling ourselves.” [52]. A scientist might observe a difference between conditions in their data, think they thought something similar previously, apply a difference test (e.g., a *t* test), and report a headline significant result. However, researchers rarely have perfect access to previous intentions and may have even forgotten thinking the opposite effect was plausible. Preregistration prevents this form of, often unconscious, error by providing an explicit timeline and record, as well as guarding against other forms of questionable research practices [19,29,30]. ECRs are at a particularly high risk of committing such errors due to lack of experience [53]. Preregistration also forces researchers to gain a more complete understanding of analyses (including stopping plans and smallest effect sizes of interest) and to attempt to anticipate all potential outcomes of an experiment [23]. Therefore, open science methods such as RRs can improve the quality and reliability of scientific work. As such procedures become more widely known, the gain in quality should reflect positively on ECRs who adopt them early.

**Fig 1:**
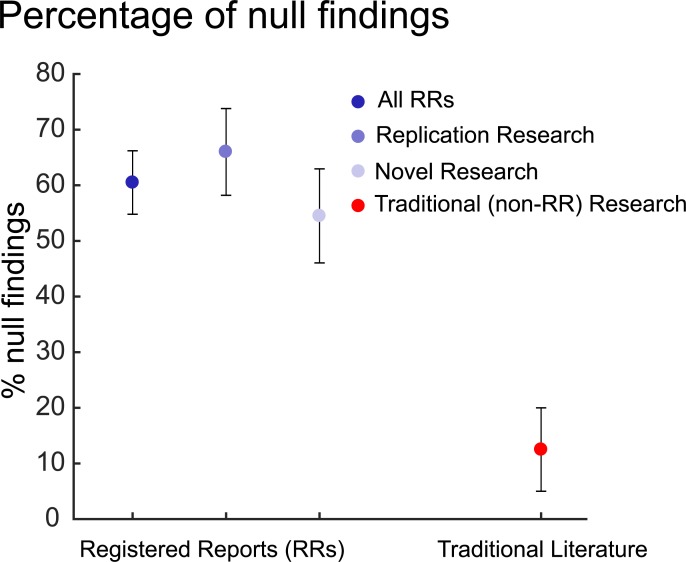
Percentages of null findings among RRs and traditional (non-RR) literature [46,47], with their respective 95% confidence intervals. In total, we extracted *n* = 153 hypotheses from RRs that were declared as replication attempts and *n* = 143 hypotheses that were declared as original research. The bounds of the confidence intervals shown for traditional literature were based on estimates (5% and 20%, respectively) of null findings that have been previously reported for traditional literature [46,47]. Data is available on the Open Science Framework (https://osf.io/wy2ek/) and in S1 Data. RR, registered report.

RRs not only guard against questionable practices but can also increase the chances of publication because they offer a path to publication irrespective of null findings. In well-designed and adequately powered experiments, null findings are often informative [23]. Furthermore, if the current incentive structure has skewed the literature toward positive findings, a higher prevalence of null findings is likely to be a better reflection of scientific enquiry. If this were the case, then we would expect more null findings in RRs and preregistrations than in the rest of the literature. To test this, we surveyed a list of 127 published biomedical and psychological science RRs compiled (September 2018) by the Center for Open Science (see S1 Text and https://osf.io/d9m6e/), of which 113 RRs were included in final analyses. For each RR, we counted the number of clearly stated, a priori, discrete hypotheses per preregistered experiment. We assessed the percentage of hypotheses that were not supported and compared it with percentages previously reported within the wider literature. Of the hypotheses we surveyed, 60.5% (see Fig 1) were not supported by the experimental data (see S1 Data and https://osf.io/wy2ek/), which is in stark contrast to the estimated 5% to 20% of null findings in the traditional literature [46,47]. The principle binomial test was applied with an uninformed prior (beta prior scaling parameters a and b set to 1), using the open software package JASP (version 0.8.5.1) [54]. Data suggested that even compared to a liberal estimate of 30% published null results, a substantially larger proportion of hypotheses was not supported among RRs (60.5% versus test value 30%, 95% confidence interval [54.7%–66.1%], *p* < 0.001; Bayes Factor = 2.0 × 10^24^). Moreover, the percentage of unsupported hypotheses was similar, if not slightly higher for replication attempts (66.0% [57.9%–73.5%]) compared with novel research (54.5% [46.0%–62.9%]) amongst the surveyed RRs. Because these comparisons are between estimates that we have surveyed and published estimates, we highlight their exploratory nature. However, these analyses suggest that RRs increase the chances for publishing null findings. Adoption of RRs might therefore reduce the chances of ECRs’ work going unpublished. Furthermore, the difference between the incidences of null findings in RRs and that of the wider literature can be interpreted as an estimate of the file drawer problem [14]. Because RRs guarantee the publication of work irrespective of their statistical significance, the ECR publishes irrespective of the study’s outcome.

A core aim of the open science movement is to make science more reliable. All the structures of open science are there to make this so. Sharing of protocols and data leads to replication, reproduction of analyses, and greater scrutiny. This increased scrutiny can also be a great motivator to ensure good quality data and analyses. Sharing data and analyses is increasingly common and expected [55], and soon we anticipate findings may only be deemed fully credible if they are accompanied by accessible data and transparent analysis pathways [56]. Instead of relying on trust, open science allows verification through checking and transparent timelines. There is also an educational aspect to this: when code and data are available, one can reproduce results presented in papers, which also facilitates understanding. More fundamentally, replication of findings is core to open science and paramount in increasing reliability, which can benefit scientists at all levels.

#### Tips

Make your work as accessible as possible and preregister experiments when suitable. If your research group lacks experience in open science practices, consider initiating the discussion. Do not be afraid of null results, but design and power experiments such that null results can be informative and register them to raise the chances of publication. When preparing preregistrations or RRs, we recommend consulting the material provided by the Center for Open Science [57]. Be aware of the additional time and power demands preregistered and RR experiments can require.

### Benefit 2: New helpful systems

The structures developed around open and reproducible science are there to help researchers and promote collaboration [58]. These structures include a range of software tools, publishing mechanisms, incentives, and international organisations. These can help ECRs in documenting their work, improving workflows, supporting collaborations, and ultimately progressing their training.

Open science software such as web-based, version-controlled repositories like GitHub archivist and Bitbucket [59] can help with storing and sharing code. In combination with scripting formats like R markdown [60] and jupyter Python Notebooks [35], ECRs can build up well documented and robust code libraries that may be reused for future projects and for teaching purposes. Ultimately, the most thankful recipient may be yourself in a few years’ time. New open tools can aid robust and reproducible data analysis in a user-friendly way. For instance, the open-source Brain Imaging Data Structure (BIDS) application was designed to standardise analysis pipelines in neuroimaging [61]. Well commented, standardised, and documented code and data are critical for making science open and improves programs, as do the additional checks when they are shared.

Open science tools can further assist ECRs in scrutinising existing work. For instance, the open software p-curve analysis was developed in response to the skew in the literature in favour of positive results and facilitates estimating publication bias within research areas ([62]; although, see [63]). Another useful checking tool is statcheck, an R toolbox that scans documents for inconsistencies in reported statistical values [64]. Overall, these examples can guide the ECR towards becoming a more rigorous researcher and may also help them to exert some healthy self-scepticism via additional checks [51].

The open science movement also provides opportunities to access free high-quality, often standardised data. For instance, in genetics the repository Addgene [65], in neuroanatomy the Allen Brain Atlas [66], in brain imaging the Human Connectome Project [67], and in biomedicine the UK Biobank [68] present rich data sources that may be in particular useful for ECRs, who often have limited funds. Furthermore, distributed laboratory networks such as the Psychological Science Accelerator [58], which supports crowdsourced research projects, and the open consortium Enhancing Neuroimaging Genetics Through Meta Analysis (ENIGMA) [69] allow ECRs to partake in international collaborations. However, although these new open forms of collaboration are often beneficial and productive, the coordination of time lines between researchers involved and expectations of contributions can be challenging and require clear and open communication.

ECRs who work with existing data sets can also benefit from new publishing formats such as secondary RRs and exploratory reports [32]. These allow preregistration of hypotheses and analysis plans for data that have already been acquired. Although still under development, exploratory reports are intended for situations in which researchers don’t have strong a priori predictions and in which authors agree to fully share data and code [32]. Similar to RRs, they are outcome independent in terms of statistical significance, and publication is based on transparency and an intriguing research question. As such, this format may present an entry to preregistration for ECRs that can help build expertise in open science methods.

There is a spectrum of open science practices and tools at researchers’ disposal. These range from making data publicly available right through to fully open RRs. Generally, researchers should be encouraged to adopt as much as possible, but one should not let the perfect be the enemy of the good. Some research questions are exploratory, may be data driven, or are iterative, which may be less well suited to preregistration. Preregistration also presents problems for complex experiments, because it can be difficult to anticipate all potential outcomes. There are also often constraints on when and if data can be made available, such as anonymization. Dilemmas also arise when elegant experimental designs are capable of probing both confirmatory and exploratory questions, in which it is recommended that only confirmatory aspects are preregistered.

#### Tips

Make use of new tools that facilitate sharing and documenting your work efficiently and publicly. Think about whether your research question can be addressed with existing, open data sets. Free training options in open science methods are growing [30]; try to make use of them. Making data and materials, such as code, available is a relatively low-cost entry into open science. ECRs should be encouraged to adopt as many open practices as possible but select the methods that fit their research question with feasibility in mind.

### Benefit 3: Investment in your future

Putting more of your work and data in the public domain is central to open science and increases ECRs’ opportunities for acknowledgment, exchange, collaboration, and advancement [43,70]. It also renders preclinical and translational research more robust and efficient [71] and can accelerate the development of life-saving drugs, for instance, in response to public health emergencies [70,72]. Reuse of open data can lead to publications [43], which may not have happened under closed science [73]. ECRs can receive citations for data alone when stored at public repositories such as the Open Science Framework [74,75], and articles with published open data receive more citations than articles that don’t share data [76]. Preprints and preregistrations are also citable and appear to increase citation rates [27]. As such, ECRs who make use of these open science methods can accumulate additional citations early on and thereby evidence the impact of their work [77].

Moreover, it has also been highlighted that authors may receive early media coverage based on preprints [27], which we see confirmed in our experience with a preprint based on an earlier version of this article [78,79]. Openness in science can even promote equality by making resource-costly data or rarely available observations accessible to a wider range of communities [74,75]. In theory, data sharing increases the longevity and therefore utility of data, whereas in closed science, data usability declines drastically over time [80]; although it is worth noting that this particular advantage is negated by inadequate data documentation [8]. More generally, with open data, it's open to anyone: in order to access, to use, and to publish using open data, one doesn't need a big grant, and therefore open science can facilitate widened participation and diversity for ECRs. In short, open science should improve the quality of work and get researchers recognised for their efforts. These benefits apply to individual career progression but also benefit science in general and thus may create a virtuous cycle.

Beyond academia, working reproducibly should put ECRs in a better career position. For instance, reproducibility has also been raised as a major concern in industrial contexts such as in software development [81], as well as in industrial biomedical and pharmaceutical research [82]. Therefore, for ECRs considering a career transition towards industry, adopting open science methods and reproducible research practices might allow them to stand out. This advantage may outweigh possible disadvantages (e.g., a shorter publication list) when it comes to career paths outside of academia [83].

Early adoption of open and reproducible methods is an investment in the future and can put researchers ahead of the curve. In the wake of numerous failed replications of previous research, employers and grant funders increasingly see open and reproducible science as part of necessary requirements and heavily encourage their adoption [51]. Recently funders have also offered funding specifically for replications and open science research projects. Some have called for data sharing [84], which is becoming a requirement for a range of biomedical journals [85]. Open access publishing has seen a rapid increase in uptake, rising by the factor of 4 to 5 between 2006 and 2016 [27], with several journals rewarding open science efforts [46]. Initiatives by leading scientific bodies demonstrate that the need for an open science culture is starting to be recognised, increasingly desired, and should become the norm [18,19,27,30,38,39]. In this spirit, the Montreal Neurological Institute as a leading neuroscience institution has recently declared itself to be a fully open science centre [86]. Other examples are the universities LMU München and Cologne (Germany) as well as Cardiff (UK) that have recently asked some candidates applying for positions in psychology to provide a track record of open science methods. Therefore, adoption of open science practices is likely to have career benefits and to grow, especially as it is a one-way street: once adopted it is very hard to revert to a traditional approach. For example, once the distinction between confirmatory and exploratory research is understood and implemented, it is difficult to unknow [4].

#### Tips

Early adoption of open science practices, which can be evidenced, will likely confer career advantages in the future. Explore opportunities for open science collaborations in consortia or research networks and connect with others to build a local open science community. Look out for open science funding opportunities, which are increasingly available. Consider the level of open science conducted when deciding where to work. Where possible, support open science initiatives.

## Conclusion

Overall, we believe open methods are worthwhile, positive, necessary, and inevitable but can come at a cost that ECRs would do well to consider. We have summarised 3 main benefits that the ECR can gain when working with open science methods and, perhaps more importantly, how open science methods allow us to place greater faith in scientific work. We also emphasize that there are obstacles, particularly for the ECR. The adoption of open practices requires a change in attitude and productivity expectations, which need to be considered by academics at all levels, as well as funders. Yet, taken together, we think that capitalising on the benefits is a good investment for both the ECR and science and should be encouraged where possible. A response to pervasive failures to replicate previous research makes the transition to open science methods necessary, and despite the challenges, early adoption of open practices will likely pay off for both the individual and science.

## Supporting information

S1 Text A description of which criteria were applied to identify discrete hypotheses, how it was established whether hypotheses were supported by data or remained inconclusive (i.e., how data was binarized), and which binominal test was carried out. (DOCX)

S1 Data A list of RRs that were surveyed as well as the data (i.e., count of hypotheses) that were used to calculate percentages (see also S1 Text) that are presented in Fig 1. RR, registered report.
(XLSX)

## Abbreviations

ECR: early career researcher
HARKing: hypothesizing after the results are known
REF: Research Excellence Framework
RR: registered reports
