# Correction: Open science challenges, benefits and tips in early career and beyond

**DOI:** 10.1371/journal.pbio.3000587

**Published:** 2019-12-06

**Authors:** Christopher Allen, David M. A. Mehler

There is an error in the reference citations in Table 1. Reference 24 is incorrectly cited; the correct reference should be 26.

The citation of references 46 and 47 in the section 'Benefit 1: Greater faith in research' (page 6 of PDF) and in the Fig 1 legend is inappropriate. The correct references are:

Fanelli D. Negative results are disappearing from most disciplines and countries. Scientometrics. 2012;90: 891–904. doi:10.1007/s11192-011-0494-7

Cristea IA, Ioannidis JPA. P values in display items are ubiquitous and almost invariably significant: A survey of top science journals. PLoS One. 2018; 1–15. doi:10.1371/journal.pone.0197440

The citations of reference 46 in the sections 'Challenge 3: Incentive structure isn’t in place yet' (page 5 of PDF) and 'Benefit 3: Investment in your future' (page 9 of PDF) are correct.

The citation of reference 47 in the section 'Challenge 3: Incentive structure isn’t in place yet' (page 5 of PDF) is correct.

The URL associated with reference 9 is incorrect. The correct URL is: https://www.sciencedirect.com/science/article/pii/B9780128161791000104

There is an error in the reference 31 and in its hyperlink. The correct reference and hyperlink are:

Kerr N. HARKing: -hypnothesizing after the results are known. Personal Soc Psychol Rev. 1998;2: 196–217.

https://doi.org/10.1207/s15327957pspr0203_4

The hyperlinked URL of reference 38 directs to the preprint version of the article. The URL of the published paper is: https://journals.sagepub.com/doi/full/10.1177/2515245918776632?casa_token=UUzRegXa2WAAAAAA%3AVhPQg9K4OwvuPW64CnGNR5EUacSc6Obb_G_P4KBFq2wfbv3ayuz9kBxIWBcYE_Uihjb4JysqtRQJ

The URL associated with reference 62 contains an error. The correct URL is: https://papers.ssrn.com/sol3/papers.cfm?abstract_id=2256237
