# Supplementary material for: Open science challenges, benefits and tips in early career and beyond
Source: PLoS Biol. 2019 May 1;17(5):e3000246. doi: 10.1371/journal.pbio.3000246 (PMC6513108; doi:10.1371/journal.pbio.3000246)
Supplement: S1 Text — (DOCX) [file pbio.3000246.s001.docx]

**Hypothesis count methodology for Allen & Mehler - Open Science challenges, benefits and tips in early career and beyond**

The aim of this analysis was to count the percentage of hypotheses being supported and when they were not, within registered reports, in order to allow for comparison to levels published in the literature.

A list of published registered report has been complied (September 2018) by the Open Science Framework (OSF): <https://www.zotero.org/groups/479248/osf/items/collectionKey/KEJP68G9/itemPage/3/order/dateModified/sort/desc>

All items on the list were considered for inclusion into the count. Manuscripts were excluded if, no preregistered experiment was reported, or, if no reference to a preregistered methodology and analysis plan was documented, unless the journal or the study indicated that it was a (pre-)registered report. Inclusion in the count was as follows: For each registered report, we counted the number of clearly stated *a priori*, discrete, hypotheses that were pre-registered per experiment (e.g. 3 hypotheses in [1]). Studies could contain multiple independent experiments that were counted separately (e.g. 2 studies in [2]). Supporting, supplementary or manipulation check hypotheses were not counted. We did not distinguish between absence of evidence and support for the null as this distinction was neither made in the majority of the registered reports we had analysed, nor in previously published estimates of null findings to which we compared our findings [3-4]. Our main analysis compared the percentage of counted unsupported hypotheses of all hypotheses counted to the percentage of null findings previously published, using a frequentist and Bayesian binominal test and a test-value of 0.3. For additionally information, replications and novel research were delineated within the registered reports surveyed. Count allocation data is available at https://osf.io/wy2ek/.

1. Frenken, M., & Berti, S. (2018). Exploring the switching of the focus of attention within working memory: A combined event-related potential and behavioral study. *International Journal of Psychophysiology*, *126*, 30–41. https://doi.org/https://doi.org/10.1016/j.ijpsycho.2018.01.012

2. Calin-Jageman, R. J., & Caldwell, T. L. (2014). Replication of the Superstition and Performance Study by. *Social Psychology*, *45*(3), 239–245. https://doi.org/10.1027/1864-9335/a000190

3. Cristea, I. A., & Ioannidis, J. P. A. (2018). P values in display items are ubiquitous and almost invariably significant: A survey of top science journals. *PLOS ONE*, *13*(5), e0197440. Retrieved from https://doi.org/10.1371/journal.pone.0197440

4. Fanelli, D. (2012). Negative results are disappearing from most disciplines and countries. *Scientometrics*, *90*(3), 891–904. https://doi.org/10.1007/s11192-011-0494-7
